# Microstructure and Mechanical Properties of Composites Obtained by Spark Plasma Sintering of Ti_3_SiC_2_-15 vol.%Cu Mixtures

**DOI:** 10.3390/ma15072515

**Published:** 2022-03-29

**Authors:** Rui Zhang, Biao Chen, Fuyan Liu, Miao Sun, Huiming Zhang, Chenlong Wu

**Affiliations:** 1 School of Mechanical Engineering, Chengdu University, Chengdu 610106, China; 17852583676@163.com (M.S.); zhanghuiming@stu.cdu.edu.cn (H.Z.); 2 State Key Laboratory of Solid Lubrication, Lanzhou Institute of Chemical Physics, Chinese Academy of Sciences, Lanzhou 730000, China; 3 Sichuan Province Engineering Technology Research Center of Powder Metallurgy, Chengdu University, Chengdu 610106, China; 4 School of Mechanical Engineering, Xinjiang University, Urumqi 830000, China; chenbiao@stu.xju.edu.cn (B.C.); w996940130@163.com (C.W.); 5 School of Chemical Engineering and Materials, Changzhou Institute of Technology, Changzhou 213032, China

**Keywords:** MAX phase, SPS, Ti_3_SiC_2_/Cu composites, sintering characteristic

## Abstract

Method of soft metal (Cu) strengthening of Ti_3_SiC_2_ was conducted to increase the hardness and improve the wear resistance of Ti_3_SiC_2_. Ti_3_SiC_2_/Cu composites containing 15 vol.% Cu were fabricated by Spark Plasma Sintering (SPS) in a vacuum. The effect of the sintering temperature on the phase composition, microstructure and mechanical properties of the composites was investigated in detail. The as-synthesized composites were thoroughly characterized by scanning electron micrography (SEM), optical micrography (OM) and X-ray diffractometry (XRD), respectively. The results indicated that the constituent of the Ti_3_SiC_2_/Cu composites sintered at different temperatures included Ti_3_SiC_2_, Cu_3_Si and TiC. The formation of Cu_3_Si and TiC originated from the reaction between Ti_3_SiC_2_ and Cu, which was induced by the presence of Cu and the de-intercalation of Si atoms Ti_3_SiC_2_. OM analysis showed that with the increase in the sintering temperature, the reaction between Ti_3_SiC_2_ and Cu was severe, leading to the Ti_3_SiC_2_ getting smaller and smaller. SEM measurements illustrated that the uniformity of the microstructure distribution of the composites was restricted by the agglomeration of Cu, controlling the mechanical behaviors of the composites. At 1000 °C, the distribution of Cu in the composites was relatively even; thus, the composites exhibited the highest density, relatively high hardness and compressive strength. The relationships of the temperature, the current and the axial dimension with the time during the sintering process were further discussed. Additionally, a schematic illustration was proposed to explain the related sintering characteristic of the composites sintered by SPS. The as-synthesized Ti_3_SiC_2_/Cu composites were expected to improve the wear resistance of polycrystalline Ti_3_SiC_2_.

## 1. Introduction

As a typical ternary layered MAX phase, Ti_3_SiC_2_ exhibited combined characteristics of both metals and ceramics. It possessed good machinability, good electrical and thermal conductivity, good ductility, thermal stability, oxidation resistance, etc. [1,2,3,4]. It has potential for applications in electro friction materials, novel structural/functional ceramic materials and high-temperature lubricating materials.

The crystalline structure of Ti_3_SiC_2_ can be described as a sandwich structure: a layer of Si and the twin boundary of TiC. The chemical bonding between the Si atom and the other atoms, such as Ti and C, was to a certain extent relatively weak compared to the strong Ti-C bonding [5,6]. Based on its unique structure, Ti_3_SiC_2_ was chemically reactive when it contacted with metals at high temperatures. Li et al. [7] fabricated Ti_3_SiC_2_/Ni and Ti_3_SiC_2_/Co through vacuum sintering. It was found that the metals (Ni and Co) were prone to aggregating towards their surfaces due to the poor wettability between Ti_3_SiC_2_ and Ni or Co. Gu et al. [8] investigated the possibility of fabricating Ti_3_SiC_2_-Ti composites. They discovered that the as-obtained composites were mainly composed of Ti_3_SiC_2_, TiC_x_, Ti_5_Si_3_, and TiSi_2_. It was inferred that the decomposition of Ti_3_SiC_2_ was owing to the de-intercalation of Si and the separation of carbon from Ti_3_SiC_2_. Gu et al. [9] studied the reactions between Ti_3_SiC_2_ and Al in the temperature range of 600–650 °C, and they found that besides Ti_3_SiC_2_ and Al, new phases Al_3_Ti, Al_4_SiC_4_ and Al_4_C_3_ were generated, the formation of which relied on the time, temperature and the relative amount of Al and Ti_3_SiC_2_. Kothalkar et al. [10] synthesized NiTi-Ti_3_SiC_2_ composite, and they discovered that the composites showed higher damping up to applied stress of 200 MPa. For example, the energy dissipation of the composite was thirteen times larger than pure Ti_3_SiC_2_ and two times larger than pure NiTi.

Except for the Ti_3_SiC_2_-metals composites described above, researchers concentrated on the investigation of Ti_3_SiC_2_/Cu composites. Dang et al. [11] fabricated Ti_3_SiC_2_/Cu composites with different contents of Cu by mechanical alloying and spark plasma sintering. They inferred that the presence of Cu leads to the decomposition of Ti_3_SiC_2_ to form TiC_x_, Ti_5_Si_3_C_y_, Cu_3_Si, and TiSi_2_C_z_. In another paper, Dang et al. [12] synthesized Ti_3_SiC_2_/Cu/Al/SiC composites by powder metallurgy/spark plasma sintering and found that the addition of Al could inhibit the decomposition of Ti_3_SiC_2_. Lu et al. [5] found chemical reaction between Cu and Ti_3_SiC_2_ contributes to the wettability. Zhou and his colleagues [13] investigated the chemical reactions and stability of Ti_3_SiC_2_ in Cu for the Cu/Ti_3_SiC_2_ composites. They found that at low content of Ti_3_SiC_2_ or below 1000 °C, Cu (Si) solid solution and TiCx were generated, whereas at high temperature or high content of Ti_3_SiC_2_, Cu-Si intermetallic compounds, such as Cu_5_Si, Cu_15_Si_4_, and TiC_x_, were generated. In our recent publication [14], Ti_3_SiC_2_/Cu composites were synthesized by spark plasma sintering technique at various temperatures. The microstructure, composition and mechanical properties of the as-obtained composites were investigated. The results indicated that the Ti_3_SiC_2_/Cu composite sintered at 1100 °C exhibited superior mechanical properties. While these results give partial information on the reactions occurring between Ti_3_SiC_2_ and Cu, they did not permit elucidation of the entire sintering behaviors occurring during the sintering process.

In this paper, the Ti_3_SiC_2_/Cu composites were fabricated by Spark Plasma Sintering (SPS) at different sintering temperatures. The sintering behaviors of the composites were explored by the characterization of the phase composition, microstructure and mechanical properties of the composites based on the relationship of the temperature, the current and the pressure with the time during the sintering process.

## 2. Experiment

### 2.1. Samples Preparation

Ti_3_SiC_2_/Cu composites were fabricated using powder mixture of Ti_3_SiC_2_ (average particle size: 38 μm, ≥98% purity, 11 technology Co., Ltd., Jilin, China) and Cu (average particle size: 74 μm, ≥99.9% purity, Macklin Biochemical Co., Ltd., Shanghai, China). The content of Cu in Ti_3_SiC_2_/Cu composites was 15 vol.%. The mixture with designed composition was first mixed by a ball-milling machine (PMQD2LB, Nanjing Chishun Technology Development Co., Ltd., Nanjing, China) with a rotational speed of 150 rpm and a ratio of ball to powder of 3 for 6 h, then filled into a graphite die (inner diameter: Φ25 mm). Finally, it was sintered by Spark Plasma Sintering (SPS, Model Labox-350, Xinxie, Japan) at different sintering temperatures (950, 1000 and 1050 °C, respectively) under a pressure of 35 MPa in vacuum for 20 min and cooled with the furnace. The heating rate was set as follows. From the room temperature to 600 °C, the heating rate was 100 °C/min. Additionally, from 600 °C to the desired sintering temperature, the heating rate was 50 °C/min.

### 2.2. Mechanical Property

The density of the as-prepared Ti_3_SiC_2_/Cu composites was measured by the Archimedes method. Cylinder specimens with a size of φ5 mm × 12 mm were machined for compressive strength testing, which was performed on WDW-100 universal materials testing machine (Jinan Hansen Precision Instrument Co., Ltd., Jinan, China). The Vickers hardness of the composites was determined in an MHVD-50AP microhardness tester (Shanghai Jujing Precision Instrument Manufacturing Co., Ltd., Shanghai, China) at a load of 1 kg with a dwell time of 10 s.

### 2.3. Analysis

The as-synthesized Ti_3_SiC_2_/Cu composites were polished using 0.5 um polishing paste by an automatic polishing machine (AutoMetTM250, Yigong Testing and Measuring Instrument Co., Ltd. Shanghai, China) for microscopic evaluation. In order to expose the grains, the polished samples were etched using a 1:1:1 by volume HF:HNO_3_:H_2_O solution and observed under optical microscopy (MDJ-DM, Chongqing Auto Optical Instrument Co., Ltd., Chongqing, China). The compression fracture morphology of Ti_3_SiC_2_/Cu composites was observed by scanning electron microscopy (SEM, JSM-6510LA, JEOL Japan Electronics Co., Ltd., Zhaodao, Japan) equipped with energy dispersive spectroscopy (EDS). X-ray diffraction (XRD) analysis was carried out on a DX-2700B diffractometer (Dandong Haoyuan Instrument Co., Ltd., Dandong, China) with Cu Kα radiation at a scanning rate of 7.2 °/min to identify the phase composition of Ti_3_SiC_2_/Cu composites.

## 3. Results and Discussion

### 3.1. Phase Composition and Microstructure

**Figure 1:**
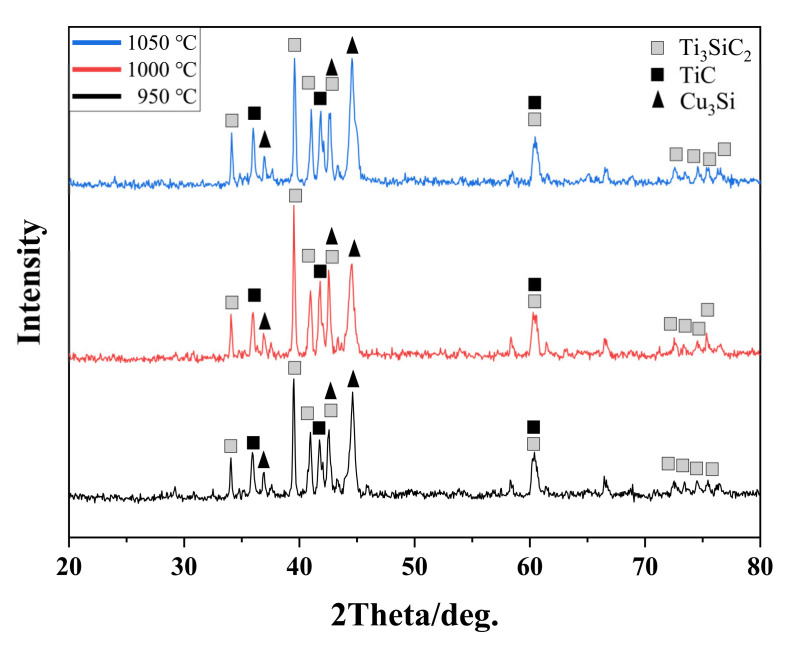
XRD patterns of Ti_3_SiC_2_/Cu composites at different sintered temperatures.

Figure 1 shows XRD patterns of the as-synthesized Ti_3_SiC_2_/Cu composites at different sintered temperatures. It was clearly observed that the as-synthesized Ti_3_SiC_2_/Cu composites were all composed of Ti_3_SiC_2_, Cu_3_Si and TiC, which was independent of the sintered temperature.

Based on the unique sandwich structure of Ti_3_SiC_2_, it exhibited high reactivity when contacting with metal phases [5,11,15,16,17,18,19]. On the one hand, the Si atoms were easily de-intercalated from Ti_3_SiC_2_. On the other hand, Si-containing solid solution or intermetallic compounds was prone to form when the metal phases contacted with Ti_3_SiC_2_. The presence of contacted metal phases accelerated the decomposition of Ti_3_SiC_2_. As for Ti_3_SiC_2_/Cu composites, Cu can form Cu (Si) solid solution or react with Si to form Cu_x_Si_y_ intermetallic compounds [18]. Therefore, the composition of Ti_3_SiC_2_/Cu composites consisted of Cu(Si) solid solution, Cu_x_Si_y_ intermetallic compounds, and TiC_z_, which were commonly examined by researchers [5,11,13].

In this study, with the change in sintered temperatures, Cu_3_Si was the only Cu-Si intermetallic compound, and TiC was the only decomposed product of Ti_3_SiC_2_. According to Guo et al. [20], when the content of Cu was less than that of Si, Cu_3_Si was the preferential product of the Cu-Si system. Because based on the binary phase diagram of Cu-Si, the content of Si in Cu_3_Si (22.2–25.2%) was the maximum among six copper silicides (Cu (Si), Cu_7_Si, Cu_5_Si, Cu_4_Si, Cu_15_Si_4_ and Cu_3_Si) [20]. This explained the reason why Cu_3_Si was the single Cu-Si intermetallic compound in our present study.

**Figure 2:**
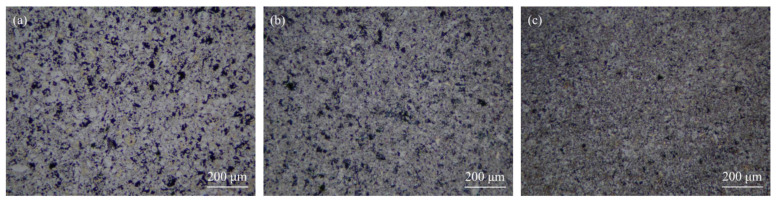
Optical micrographs of polished and etched Ti_3_SiC_2_/Cu composites sintered at 950 °C (**a**), 1000 °C (**b**) and 1050 °C (**c**).

The effect of sintered temperature on the microstructure is shown in Figure 2. As seen in Figure 2a, at 950 °C, the typical plate-like morphology of Ti_3_SiC_2_ grains was evidently inhibited by the addition of Cu. A considerable amount of Ti_3_SiC_2_ equiaxed grains appeared due to the reaction between Ti_3_SiC_2_ and Cu. Additionally, with the increase in the sintered temperature from 950 °C to 1050 °C, the Ti_3_SiC_2_ granules decreased, which was accompanied by fewer pores or holes, indicating higher reactivity of Ti_3_SiC_2_ and Cu.

**Figure 3:**
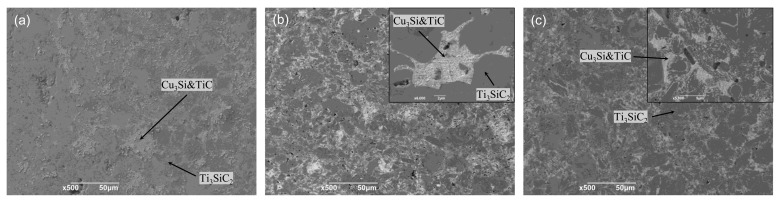
BSE micrographs of the Ti_3_SiC_2_/Cu composites sintered at (**a**) 950 °C, (**b**) 1000 °C (inset showed the structure at a higher magnification) and (**c**) 1050 °C (inset showed the structure at a higher magnification).

The back scattering electron images of polished and etched Ti_3_SiC_2_/Cu composites sintered at 950 °C, 1000 °C and 1050 °C are shown in Figure 3. As mentioned above, the main composition of Ti_3_SiC_2_/Cu composites were Ti_3_SiC_2_, Cu_3_Si and TiC. Ti_3_SiC_2_ was located at the dark grey area in Figure 3; both Cu_3_Si and TiC were located at the light grey area in Figure 3. It was clearly seen from Figure 3 that the as-formed Cu_3_Si distributed along the grain boundary of Ti_3_SiC_2,_ and it was accompanied by the formation of hard TiC particles. Moreover, with the increase in the sintered temperature, the reaction between Ti_3_SiC_2_ and Cu became more severe, resulting in the formation of Cu_3_Si with a non-negligible amount, especially at 1000 °C. The reaction mechanism between Ti_3_SiC_2_ and Cu was proposed as follows. After milling, the Cu powder is relatively evenly distributed in Ti_3_SiC_2_ powder. At elevated temperatures (for example, 900 °C), the Si atoms de-intercalated from Ti_3_SiC_2_ grains, diffused rapidly around Cu and reacted with Cu to form Cu_3_Si. Meanwhile, the original Ti_3_SiC_2_ skeleton structure transformed to TiC structure, which was concomitant with the formation of pores or holes due to the mismatch of skeletal density between Ti_3_SiC_2_ and TiC. When the sintered temperature raised (for example, 1000 °C), the de-intercalation of Si atoms from the Ti_3_SiC_2_ skeleton was accelerated by the defects (pores or holes) and high temperature. On the other hand, Cu had a tendency to melt and flow around the grain boundaries of Ti_3_SiC_2_ grains and the as-formed defects mentioned above. All these led to more violent reactions between Ti_3_SiC_2_ and Cu, causing the Ti_3_SiC_2_ grains to become smaller and smaller. This reaction process well coincided with Zhou et al. [13]. Theoretically, with the increase in temperature, the reactivity of Ti_3_SiC_2_ and Cu increased, producing more Cu_3_Si. However, when the sintered temperature reached 1050 °C, a large amount of Cu melted and released from the graphite die, leading to the loss of Cu. As seen in Figure 3, visually, the content of Cu_3_Si was largest at 1000 °C, not at 1050 °C.

**Figure 4:**
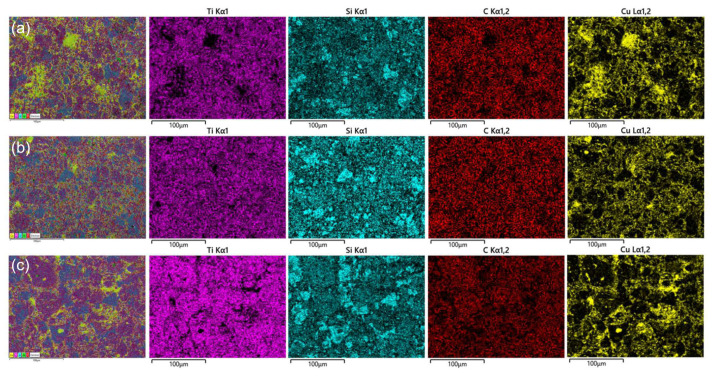
Mapping of elemental distribution for Ti_3_SiC_2_/Cu composites sintered at 950 °C (**a**), 1000 °C (**b**) and 1050 °C (**c**).

Figure 4 illustrates the mapping of elemental distribution for Ti_3_SiC_2_/Cu composites sintered at different temperatures. It was irrefutable evidence for the explanation of the sintering effect of the composites. At 950 °C, the Cu considerably aggregated in the Ti_3_SiC_2_/Cu composites (see Figure 4a). At this temperature, a solid–solid sintering process occurred. The agglomeration of Cu relied on the uniformity of its distribution in Ti_3_SiC_2_ during ball milling. As we know, ball milling cannot avoid material agglomeration. At 1000 °C, it was obviously observed that Cu was relatively evenly distributed in the Ti_3_SiC_2_/Cu composites (see Figure 4b). It was speculated that solid–liquid sintering process took place at 1000 °C. The appropriate flowability of the quasi-liquid Cu contributed to not only its reaction with Si atoms but also its uniform distribution in the composites. At 1050 °C, the Cu melted and rapidly flowed around the Ti_3_SiC_2_ grains, leading to the relatively obvious aggregation of Cu (see Figure 4c). Moreover, the release of liquid Cu during sintering caused the loss of Cu to a certain extent. Therefore, it was concluded that the optimal sintering temperature was 1000 °C for the Ti_3_SiC_2_/Cu composites in our study.

### 3.2. Mechanical Property

**Figure 5:**
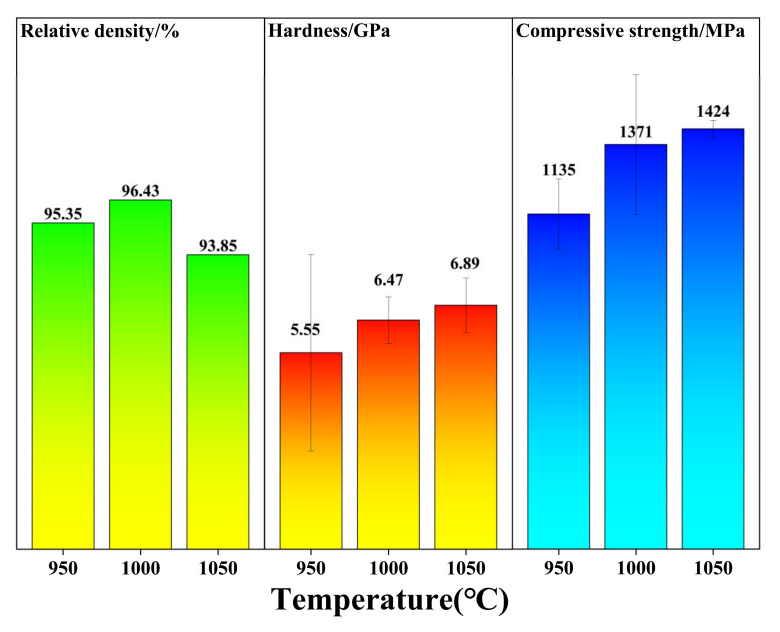
The variation in relative density, hardness and compressive strength of the Ti_3_SiC_2_/Cu composites versus the sintered temperature.

The variation in relative density, hardness and compressive strength of the Ti_3_SiC_2_/Cu composites with the sintered temperature are shown in Figure 5. As seen in Figure 5, the relative density of the Ti_3_SiC_2_/Cu composites sintered at 950 °C was 95.35%, which was slightly lower than that sintered at 1000 °C (96.43%). However, the relative density of the composite sintered at 1050 °C was 93.85%, which was the lowest among the three samples. The higher relative density of the Ti_3_SiC_2_/Cu composites sintered at 1000 °C was attributed to the synergetic effect of high temperature, high pressure and pulse current during the sintering process. The most important thing was that the quasi-liquid Cu possessed proper mobility, which was beneficial for the densification of the composites as well. At 1050 °C, its lowest relative density was also related to the flowability of Cu. In such circumstances, Cu flowed easily and released rapidly, causing the aggregation of Cu (see Figure 4c) and the loss of Cu (see Figure 3c).

As shown in Figure 5, both the hardness and the compressive strength of the Ti_3_SiC_2_/Cu composites increased with the increase in the sintered temperature. It was clearly seen that the deviation of the hardness of the composites sintered at 950 °C was the highest among the three samples, which originated from the agglomeration of Cu in the composites (see Figure 3a and Figure 4a). Compared with the polycrystalline Ti_3_SiC_2_ (5.5 GPa) [21], the higher hardness of the composites at different temperatures came from the formation of hard TiC product, which was detected by XRD analysis (see Figure 1).

**Figure 6:**
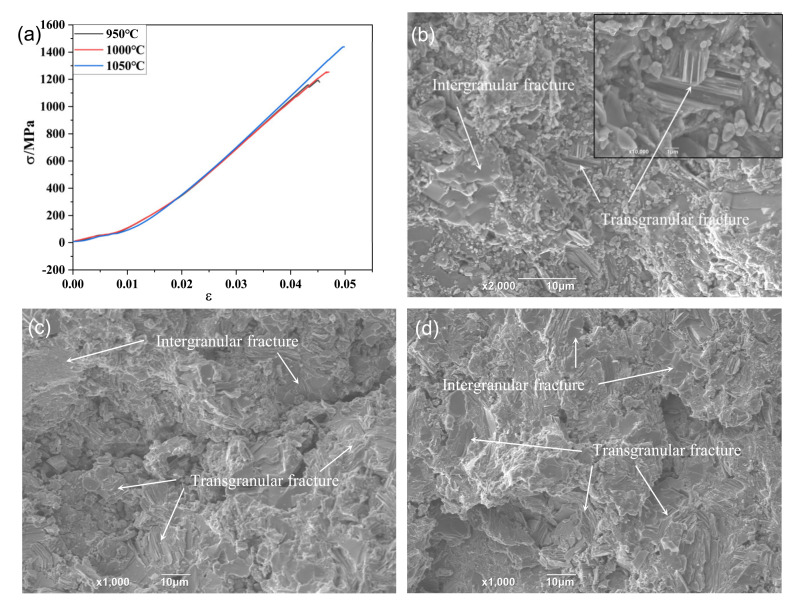
(**a**) Compressive stress–strain curve of Ti_3_SiC_2_/Cu composites, and the fracture morphology of Ti_3_SiC_2_/Cu composites sintered at (**b**) 950 °C (inset showed the structure at a higher magnification), (**c**) 1000 °C and (**d**) 1050 °C.

Additionally, in comparison with the polycrystalline Ti_3_SiC_2_, the compressive strength of the composites sintered at different temperatures exhibited an equivalent or higher value [22], which was due to the appropriate reaction between Ti_3_SiC_2_ and Cu. As mentioned above, during the sintering process, the de-intercalation of Si from Ti_3_SiC_2_ and thereafter the dissolution of it in the liquid Cu phase led to the formation of TiC and Cu_3_Si. Moreover, the Cu_3_Si is uniformly distributed along the grain boundary of Ti_3_SiC_2_. The as-obtained fine TiC and Cu_3_Si grains were uniformly distributed along the boundary of Ti_3_SiC_2_ grains, which was a benefit for the higher compressive strength of the composite. As seen in Figure 6a, the Ti_3_SiC_2_/Cu composites showed a brittle fracture character, which was identical with polycrystalline Ti_3_SiC_2_. From the compression fracture morphology of the composites (see Figure 6b–d), it was apparently seen that both the intergranular fracture and transgranular fracture were present on the compression fracture surface of the composites sintered at different temperatures. It indicated that the fracture mode of the composites was independent of the sintered temperature.

### 3.3. Sintering Behaviors of the Ti_3_SiC_2_/Cu Composites

**Figure 7:**
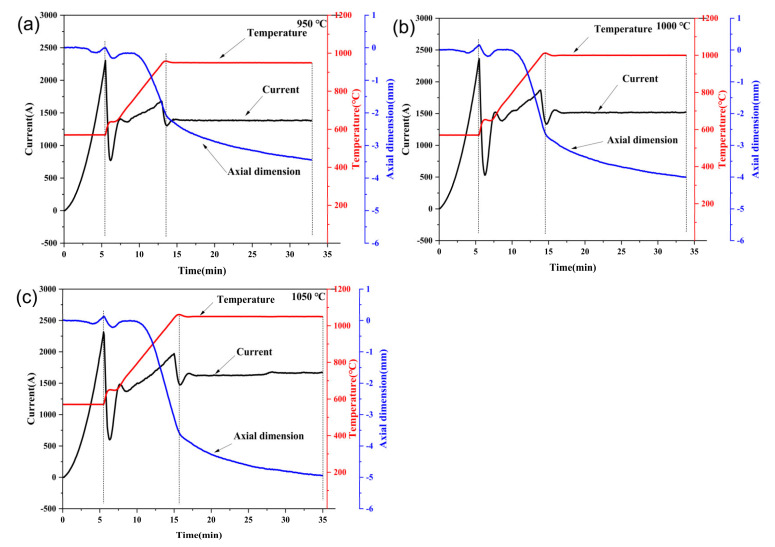
The relationship of the temperature, the current and the dimensional change with sintering time for the Ti_3_SiC_2_/Cu composites sintered at (**a**) 950 °C, (**b**) 1000 °C and (**c**) 1050 °C.

It is instructive to explain the sintering process of the Ti_3_SiC_2_/Cu composites at different temperatures. The relationships of temperature, current and axial dimension with time is illustrated in Figure 7. In comparison, although the temperature difference for the three sintering temperatures was the same (950–1000 °C and 1000–1050 °C), the change in the axial dimension was different. The change in the axial dimension for 950–1000 °C was obviously lower than that for 1000–1050 °C.

The profile of temperature, current and axial dimension with time was insensitive with the sintering temperature (see Figure 7a–c). We took 1000 °C as an example to elaborate the related connections among the temperature, the current, the axial dimension and time. As seen in Figure 7b, from room temperature to 600 °C (Note: without monitoring temperature by the infrared thermometer), the heating rate was fast (approximate 100 °C/min) in order to remove moisture to dry powder completely, thus the current increased rapidly to 2300 A. During this period, the decrease in the axial dimension was due to the action of pressure and the softening due to the heating, and the first maximum of the axial dimension at about 5 min resulted from the thermal expansion and contraction. Hereafter, from 600 to the sintering temperature (for example, 1000 °C), the heating rate was set as 50 °C/min; therefore, the current rapidly adjusted to a low value (about 750 A) and then increased at a proper rate to make sure the sintering temperature intelligently controlled. At the same time, the axial dimension was controlled by a comprehensive impact of pressure, current and the thermal expansion and contraction, and it kept relatively stable before 10 min, then it remarkably decreased from 800 °C to the sintering temperature (for example, 1000 °C). The rapid decrease in the axial dimension was mainly attributed to the densification effect due to the continuous pressure at the sintering temperature and possibly due to the reaction between Ti_3_SiC_2_ and Cu. Finally, in the holding period, both the temperature and the current were constant, and the axial dimension slowly decreased as a result of the further densification. Additionally, an extensive reaction between Ti_3_SiC_2_ and Cu was partly beneficial for the decrease in the axial dimension.

**Figure 8:**
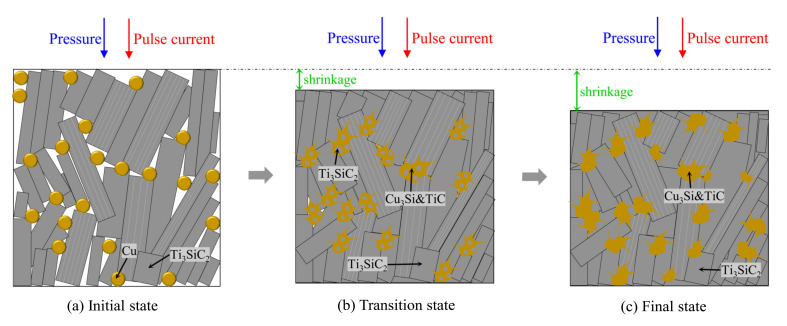
Proposed schematic illustration showing the sintering process of the Ti_3_SiC_2_/Cu composites: (**a**) initial state, (**b**) transition state and (**c**) final state.

The proposed sintering process of the Ti_3_SiC_2_/Cu composites is shown in Figure 8. In the initial state (see Figure 8a), Ti_3_SiC_2_ and Cu were relatively uniformly distributed in the graphite die, which corresponded to the state of the first 10 min in Figure 7. During the initial stage, there was no reaction between Ti_3_SiC_2_ and Cu, and the softening of the powder and the thermal expansion and extraction were due to the action of the current and the pressure. In the transition state (see Figure 8b), under the synergetic effect of the current, temperature and pressure, Cu locally melted and was extruded into the grain boundary of Ti_3_SiC_2_.

On the other hand, Ti_3_SiC_2_ grains underwent plastic deformation under the same condition. All these factors increased the contact area of Ti_3_SiC_2_ and Cu. Additionally, the de-intercalation of Si atoms from Ti_3_SiC_2_ and its diffusion contributed to the reaction between Ti_3_SiC_2_ and Cu, leading to the formation of Cu_3_Si and TiC, which was distributed along the grain boundary of Ti_3_SiC_2_ grains. Moreover, the quasi-liquid Cu diffused into the inner part of Ti_3_SiC_2_ grains through the holes or pores produced by the mismatch of skeleton density of the original Ti_3_SiC_2_ and the as-formed TiC and reacted further with the Si atoms de-intercalated from the inner Ti_3_SiC_2_ grains. For the Ti_3_SiC_2_ grain surrounded by Cu, its surface was continuously transformed into TiC, which was accompanied by the formation of Cu_3_Si. Accordingly, the grain consisting of Cu_3_Si and TiC, which was embedded by Ti_3_SiC_2_, was expected (see the inset of Figure 3b and Figure 8b). Therefore, the grain size of Ti_3_SiC_2_ became smaller and smaller, which was inconsistence with the result in Figure 2. This state (the transition state in Figure 8b) corresponded to the rapid decrease in the axial dimension in Figure 7. In the final state (see Figure 8c), the reaction of Ti_3_SiC_2_ and Cu continued, and the pores were filled by the product, densifying the composites. This final state corresponds to the holding period in Figure 7. Consequently, the Ti_3_SiC_2_/Cu composites with superior mechanical properties were obtained at 1000 °C for 20 min by SPS. The proposed sintering process undoubtedly inspired us to further explore the sintering of Ti_3_SiC_2_/Cu composites in detail.

## 4. Conclusions

Ti_3_SiC_2_/Cu composites were sintered by Spark Plasma Sintering (SPS) in a vacuum under a pressure of 35 MPa at 950–1050 °C. The as-synthesized composites were systematically characterized by scanning electron micrography (SEM), optical micrography (OM) and X-ray diffractometry (XRD), respectively. The results indicated that the constituent of the Ti_3_SiC_2_/Cu composites included Ti_3_SiC_2_, Cu_3_Si and TiC, which was insensitive to the sintering temperature. The reaction between Ti_3_SiC_2_ and Cu produced Cu_3_Si and TiC, the formation of which was attributed to the presence of Cu and the de-intercalation of Si atoms Ti_3_SiC_2_. OM analysis confirmed that with the increase in the sintering temperature, the Ti_3_SiC_2_ became smaller and smaller, which resulted from the more violent reaction between Ti_3_SiC_2_ and Cu. Moreover, SEM analysis demonstrated that the agglomeration of Cu in the composites limited the uniformity of the microstructure distribution of the composites, governing the mechanical behaviors of the composites. At 1000 °C, the distribution of Cu in the composites was relatively uniform; thus, the composites exhibited the highest density, relatively high hardness and compressive strength. The relationships of the temperature, the current and the axial dimension with the time during the sintering process were further discussed, and a corresponding schematic illustration was proposed to explain the related sintering behaviors of the composites sintered by SPS.

## Data Availability

Not applicable.
